# Supplementary material for: Affectation of COVID-19 pandemic on the use and abundance of wild resources in Tabasco, Mexico: A qualitative assessment
Source: PLoS One. 2024 Mar 11;19(3):e0299744. doi: 10.1371/journal.pone.0299744 (PMC10927097; doi:10.1371/journal.pone.0299744)
Supplement: S1 Appendix — (DOCX) [file pone.0299744.s001.docx]

**Appendix**

Common names of the most common species in their use and exploitation in the state of Tabasco, México

**Timber**

Oak, chakté, nance, tachicón, mimosas, cassias, jícaros, macuilís, macayo, mulato tree, ceiba, acacias, sichi, tintales, pitche, cocohite, caracolillo, huapaque, tucuy, barí amargoso, jobo, etc., icacoel sivil, la crucetilla and juncos. Mangrove for consturction and charcoal.

**Non timber**

Sabal palm, ferns and hanas, coconut palm, abanico palm, round palm, royal palm, long guano, jahuacte, anona, bambú, sandpaper tree, cocoyol, palm fasiste and brahea palm, dulcís, vines, lianas. guácimo, guano yucateco, round guano, beach grape, amaranth, day time don Diego, majagua, dwarf banana, muste, anonillo and julube; tó leaves o pantanola espadañal o cat tail, tall juncales, water jacinto, acuatic lirio, the lettuce, chintul, cow tong, hot bread, el sargazo, dormilona, pancillo, sunleave, purple and dwarft guineo, uspí, surumuflo, banana, cocoa and coffee tree.

**Flora**

Capulín, popiste, ramoncillo, several types of orchids, Tabasco pepper, pucté, majagua, water zapote, sivil, molinillo; many fruit trees like cuijinicuil, mamey, tamarind, coconut, papayo, guanábana, anona, pitahaya, nance, caimito, melocotón, corozo, chinín, avocato, marañón, guayaba, toronja, sour lemon, royal lemon, sweet and sour orange, prune, jondura, guaya, chicozapote. Herbs like chipilín, chaya, momo, perejil, epazote, ruda, toronjil, maguey, yerbabuena y cañafístola. jagua, Many chilli peppers like amashito, picopaloma, garbanzo and tubers like yuca and macal.

**Fauna**

Spider monkey, zaraguatos, racoon, squirrels, quail, ant-bear, deer, armadillo, wild pig, porcupine, tepezcuintle and wild rabbit. Many fishes like mojarras, alligator gar, charales, topenes, river clams and snailes. Reptiles like caimanes, cocodrile, turtles like hicoteas, pochitoques, guaos, chiquiguaos, frogs, salamander, and toads. Many birds like pijijes, almond duck, white and black heron, and many waterfowl that are migrants or winter visitors like ánade duck long tail, white wing teal, american zarceta and snow goose. Among the few mammals that inhabit swamps and lakes are the manatee, otter and rice rat. Mangrove fauna like crabs and shellfish such as oysters, hook mussel, limpet and crabs. At the intersection of the saline lagoons and the sea there is aquatic fauna like sábalo, pámpano, robalo and sea mojarra.
