# Supplementary material for: Affectation of COVID-19 pandemic on the use and abundance of wild resources in Tabasco, Mexico: A qualitative assessment
Source: PLoS One. 2024 Mar 11;19(3):e0299744. doi: 10.1371/journal.pone.0299744 (PMC10927097; doi:10.1371/journal.pone.0299744)
Supplement: S1 File — (PDF) [file pone.0299744.s002.pdf]

USE OR EXPLOITATION OF any WILD RESOURCE in your Location

The purpose of this questionnaire is to know if the WILD resources of Tabasco have suffered any impact during the current COVID-19 pandemic, addressing how they were used before and how are they used now.
This survey is Anonymous and its target is academic-scientific, so we need for the greatest truthfulness in your answers.
In this survey, a wild resource means for example: food plants, medicinal plants, animals for food such as turtles, iguanas, etc. that we obtain directly from the field or road, and that we do not buy in the market or other place.
This survey is aimed only at people who have made some use or exploitation of a natural resource or who knows about it.
If you have any questions or comments, at the end of the questionnaire there is a space to express them.

1. Do you have a family member at home or outside home who takes advantage of some wild resources in the countryside?

   ○ Yes

   ○ No

2. Municipality

3. Location or village

4. Gender

- Male
- Female

5. Age (yr-old)

6. BEFORE March 2020, did you or any family member use any wild resource?

- ◯ Yes
- ◯ No

7. Indicate which product(s) you obtain from the field and how frequently

	Weekly	Bi-weekly	Monthly	Few times a yer
Medicine plants	○	○	○	○
Wood, firewood, charcoal, etc.	○	○	○	○
Edible animals like armadillos, iguanas, turtles, crabs, etc.	○	○	○	○
Plants, floweres, leaves, palm leaves, etc.	○	○	○	○
Edible plants like coconuts, yuca, flowers, fruits, etc.	○	○	○	○
Other	○	○	○	○

8. How frequently was the resource used BEFORE the pandemia?

- ◯ Much
- ◯ Moderately
- ◯ Few

9. Regarding the resources you mentioned, do you think that 4 or 5 years ago the resources were?

- ◯ Abundant and easy to find
- ◯ Moderately abundant
- ◯ Rare and difficult to find

10. How intensely do you think you obtained the products before the pandemic?

- ◯ Much
- ◯ Moderetaly
- ◯ Few

11. In which months is the use of these resources carried out?

12. During the last 5 years, how many years have you taken advantage of them?

- ◯ 1
- ◯ 2
- ◯ 3
- ◯ 4
- ◯ 5

13. The resources obtained from the field are for

- ◯ home food
- ◯ Sell
- ◯ Other

14. To what extent do the natural resources mentioned help the family economy?

- ◯ It is the main economic support
- ◯ It is only a part of the family income
- ◯ It is only for family consumption
- ◯ Other

15. AFTER March 2020, did you use natural resources?

- ◯ Yes
- ◯ No

16. Indicate which product(s) is still obtained from the field and how frequently

	Weekly	Bi-weekly	Monthly	Few times a year
Medicine plants	○	○	○	○
Wood, firewood, charcoal, etc.	○	○	○	○
Edible animals like armadillo, turtle, iguana, crabs, ets.	○	○	○	○
Plants, seeds, leaves, palm leaves, etc.	○	○	○	○
Edible plants like fruits, flowers, leaves, coconut, yuca, etc.	○	○	○	○
Others	○	○	○	○

17. To what intensity do you think you have taken advantage of it during the current pandemic?

- ◯ Much
- ◯ Moderately
- ◯ Low

18. To what extent do you think the pandemic affect the use of wild resources?

- ◯ It didn't affect and we took advantage of it anyway
- ◯ It did affect and we did not take advantage of it

19. Currently you consider that the resources are

- Abundant and easy to find
- Moderately abundant
- Scarce and difficult to find

20. AFTER March 2020 these natural resources in the field are

- More abundant than BEFORE March 2020
- Equal
- Less abundant than BEFORE March 2020

21. Currently wthat do you think about the products sells during this pandemic

- Sales increased
- They stayed the same
- Sales decreased
- I do not sell

22. During this pandemic you stay at home with your family

- longer than before
- Less than before
- Equal time

23. When you leave home do you apply sanitary measures?

- ◯ Always
- ◯ Some times
- ◯ Never

24. What type of sanitary measures do you use when leaving home?

- ◯ Healthy distance
- ◯ Face mask
- ◯ Gel

25. Does the use or exploitation of natural resources require contact with more than 5 people?

- ◯ Yes
- ◯ No

26. How many members at home have got Covid?

27. Have any family member died due to COVID?

- ◯ Yes
- ◯ No

28. Additional comments that can help us to understand the effects of the pandemic on natural resources

This content is neither created nor endorsed by Microsoft. The data you submit will be sent to the form owner.

Microsoft Forms
