# Supplementary material for: Affectation of COVID-19 pandemic on the use and abundance of wild resources in Tabasco, Mexico: A qualitative assessment
Source: PLoS One. 2024 Mar 11;19(3):e0299744. doi: 10.1371/journal.pone.0299744 (PMC10927097; doi:10.1371/journal.pone.0299744)
Supplement: Attachment — Submitted filename: Response to Reviewers.docx [file pone.0299744.s004.docx]

Response letter to PONE-D-23-33512 Review

MS was revised and corrected following the two style pdf templates.

The following Ethical disclosure statement was placed at the end of the Methods section and as a supplementary file. “Here we want to declare that no formal or written consent was necessary to request from the participants because their participation consisted only in responding a 7 minutes friendly face to face survey with a short talk. Before the application of the survey only a verbal consent was request. All the participants were over 18-yr old.”

The Funding Information was amended, withdrawn from the MS and placed in the Cover Letter and online submission.

As an important issue, the editor request to reanalyze the data and to improve the statistical methods. However throughout the review, this point is never specified or a statistical procedure suggested, and then I could not define a way to make it. In such case, I would kindly request to indicate which procedures to follow instead.

Anyway, I would like to justify the statistical analysis used in the study. Since the results (Table 1, Figures 2 – 4) are percentages, which takes values within a range 0 – 100 (0 – 1.0) and do not constitute an infinitive variable, a non-parametric test like Z test is recommended instead of parametric analysis by means or medians comparison. Their Z standard errors and respective P values were obtained (pg. 13). For percentage data which came from a Likert scale (classes of abundance, impact and use) a Cronbach's alpha test was first applied to ensure the reliability of the scale classes performed. All this is commented within the Methods section (pg. 11).

The writing was revised and modified hoping is now better.

Eight out of ten suggested references, plus others Plos One references, were incorporated to the MS.

**Response to Reviewers**

Raw data was made available through a supplementary S3 file.

P values were incorporated to Table 1, and title and footnote arranged according to journal style.

Original questionnaire (link, pg. 11 of MS) and english version questionnaire (S2 file), and Consent letter were added as supplementary files.

Reviewer 2:

As suggested by the reviewer, the title was modified.

Abstract was improved as suggested.

Keywords were replaced using MESH tool.

Ten references were suggested to incorporate to the Background and eight references were included considering its pertinence. Plus this, two more recent references from Plos One were also included.

The name of study was brought in methods section at the beginning. Sorry, this recommendation was not very clear for me.

The reviewer asked to add the time duration of study. However this was already mentioned on line 291 of the MS.

The reviewer asked to please describe how the location of sampling was selected, in details. This was amended in page 14 of the revised MS.

The reviewer stated “Statistical analysis of sample data should be modified”, but there is nothing else suggesting how to improve the statistical procedures. In this way, I unfortunately could not select a different procedure to reanalyze data based on the objectives of the study, which I personally consider that these can be supported with the current statistical methods. Specifically, the paragraph from line 242 – 244 was unclear. It was rewritten.

The unique table of the MS was improved.

The reviewer stated “Refer to more updated articles on similar studies in the discussion section and also reference list.” Actually discussion section has about 10 references from 2020 to 2022, and I could only added one suitable reference from 2022 since this particular topic still remains scarcely studied and reported worldwide.

Study's strengths and limitations were improved and included at the end of the MS.

Future research directions were also improved and included at the end of the MS.

Conclusions were shortened keeping the relevant findings of the study.
